# Supplementary figures and images for: Dysfunction of Prkcaa Links Social Behavior Defects with Disturbed Circadian Rhythm in Zebrafish
Source: Int J Mol Sci. 2023 Feb 14;24(4):3849. doi: 10.3390/ijms24043849 (PMC9961154; doi:10.3390/ijms24043849)

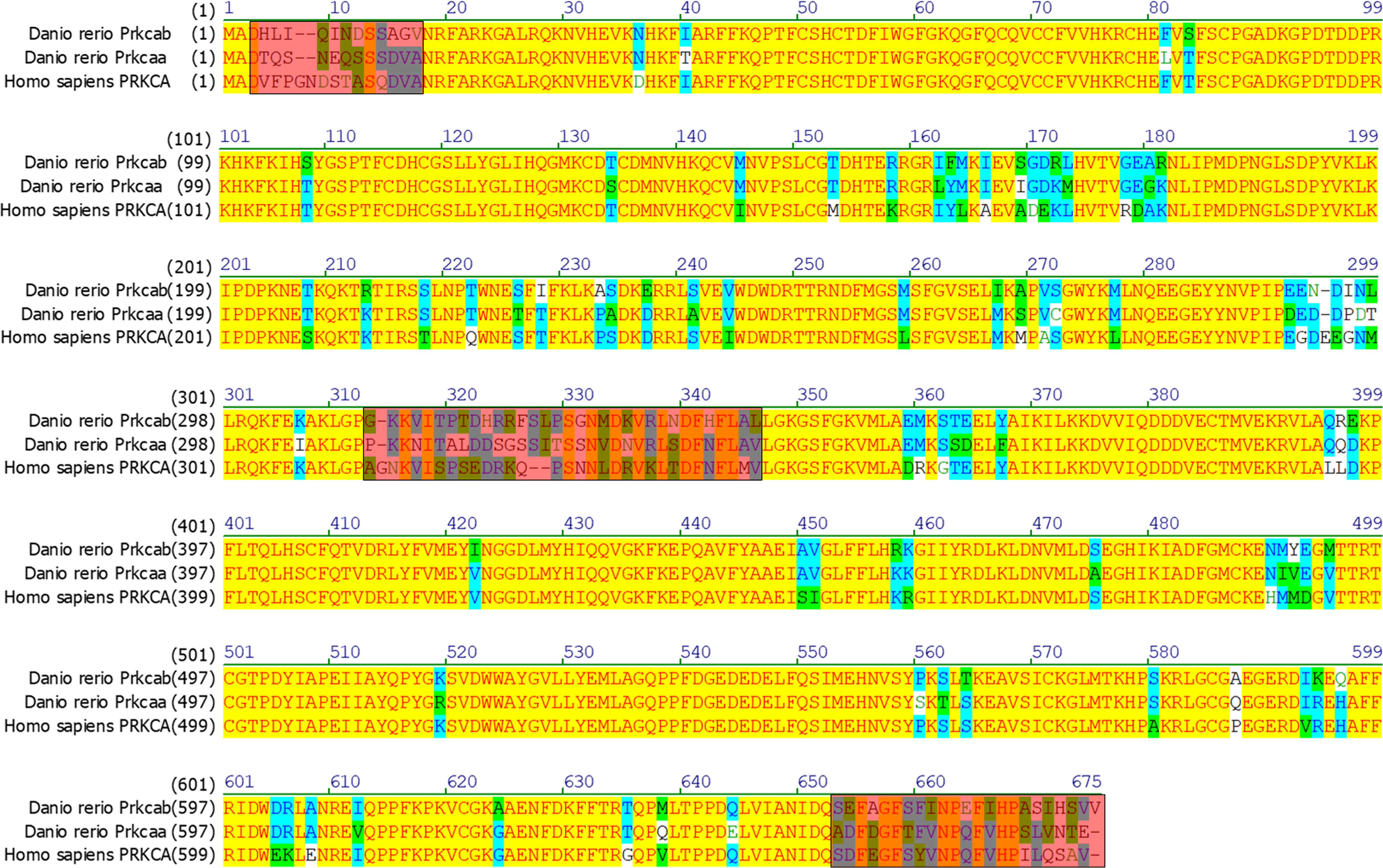

Supplement: Supplementary file 1 [file ijms-24-03849-s001.zip › Figure S1.tif]

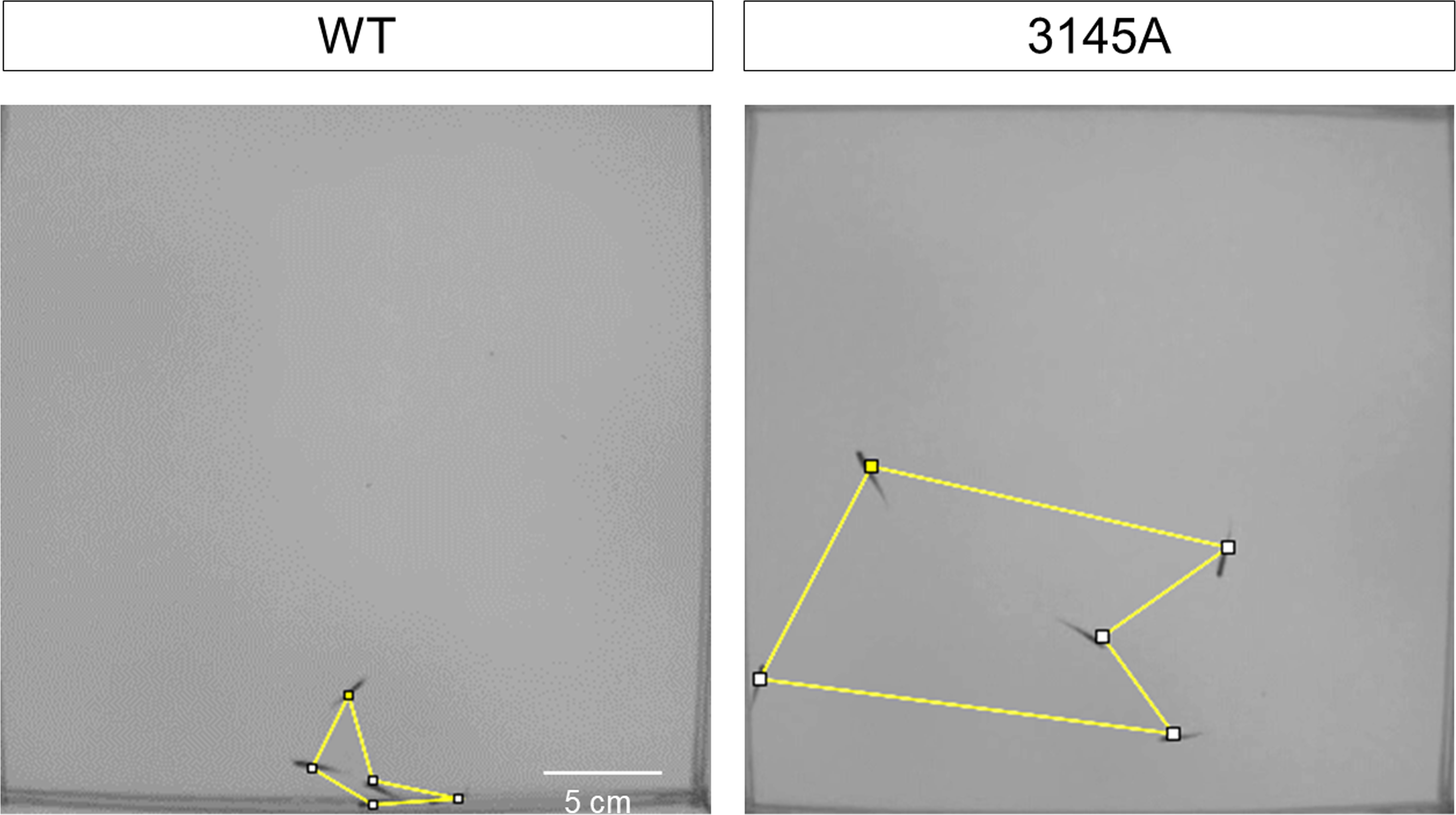

Supplement: Supplementary file 1 [file ijms-24-03849-s001.zip › Figure S2.tif]

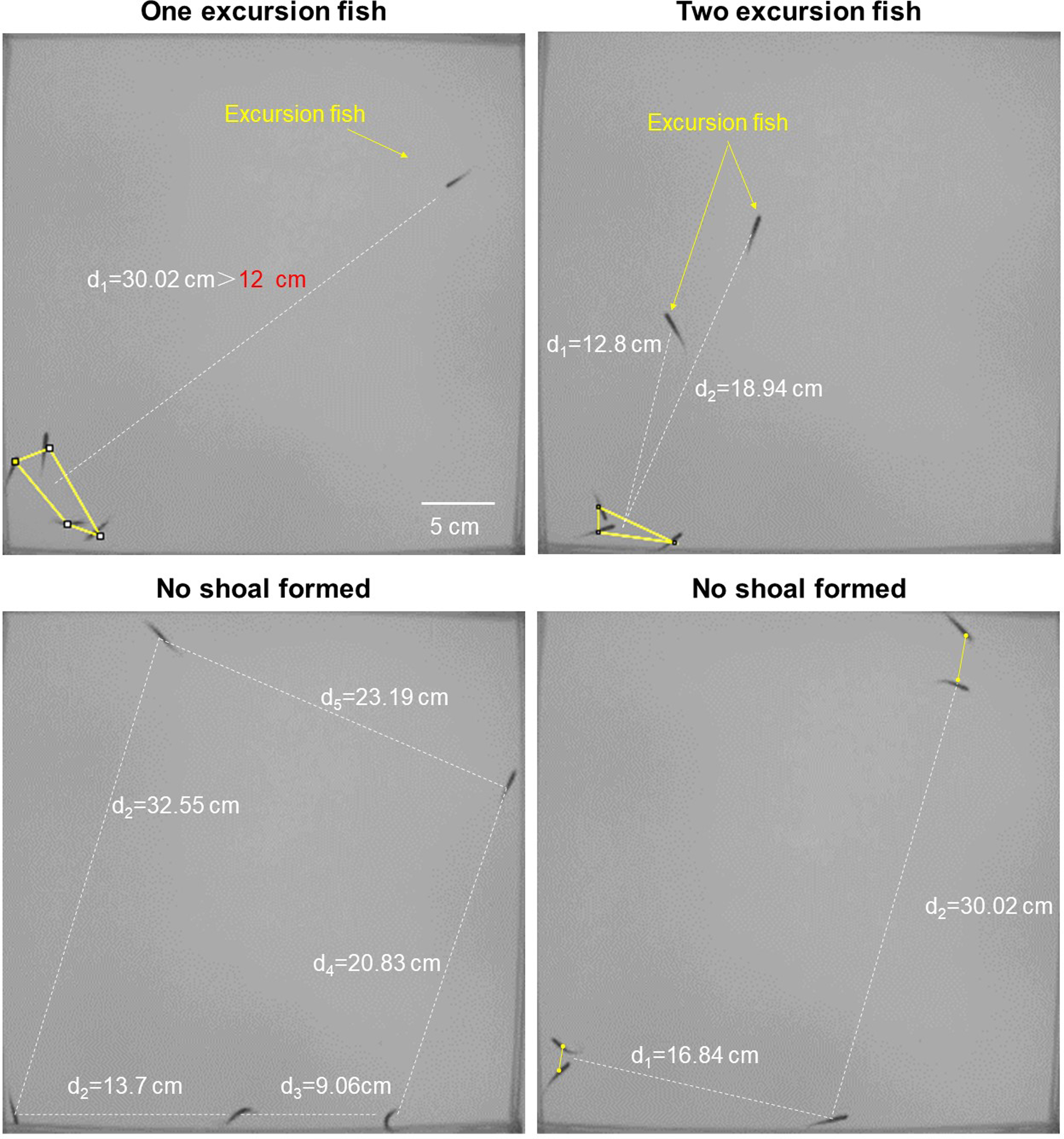

Supplement: Supplementary file 1 [file ijms-24-03849-s001.zip › Figure S3.tif]

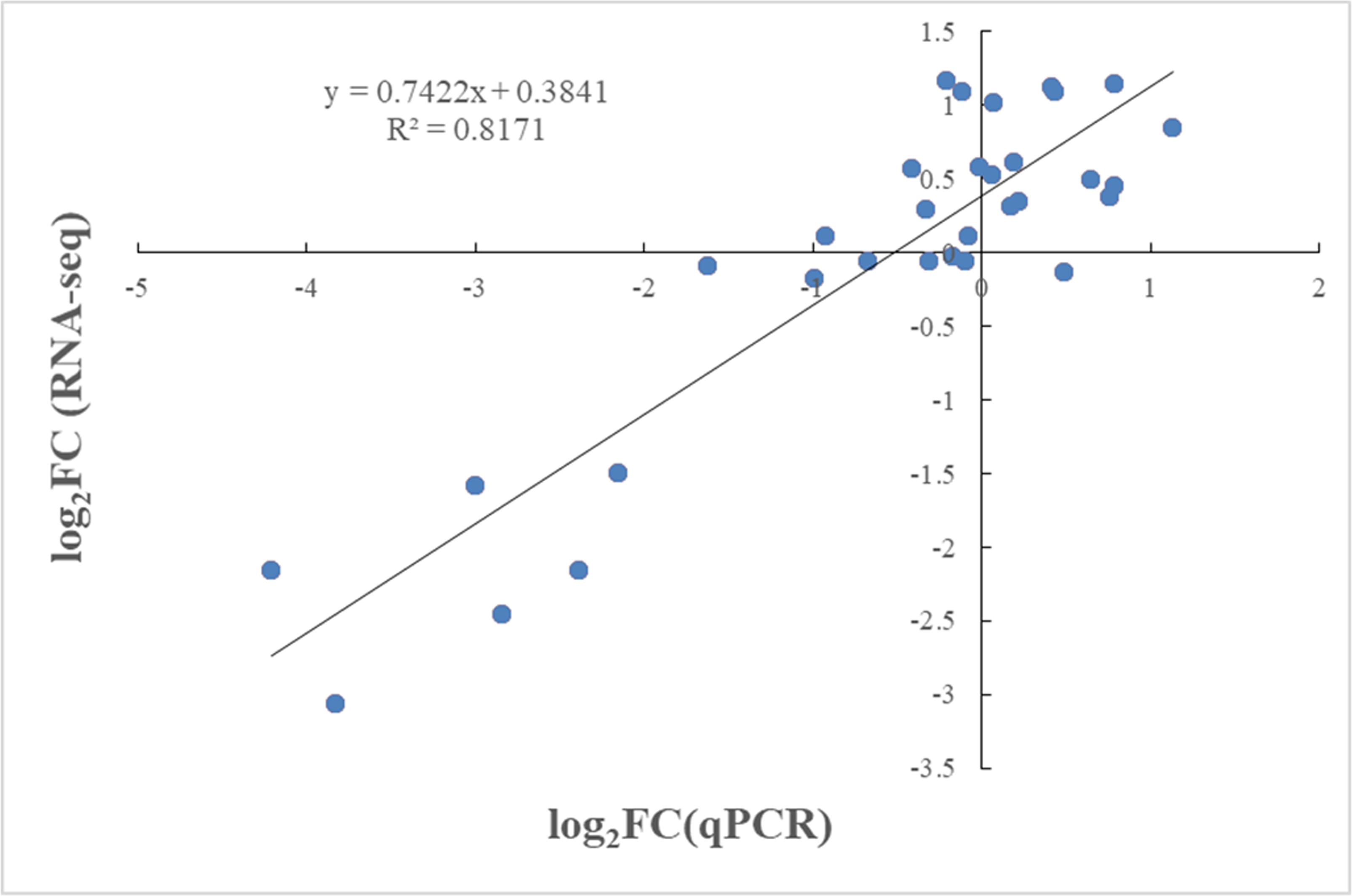

Supplement: Supplementary file 1 [file ijms-24-03849-s001.zip › Figure S4.tif]
